# Serum-urine metabolic integration via UPLC-QTOF/MS uncovers shared pathway biomarkers for cirrhosis diagnosis

**DOI:** 10.3389/fmed.2025.1646323

**Published:** 2026-02-09

**Authors:** Xiaogang Li, Runxi Wang, Hongbing Zhou, Ruixue Li, Hong Chang, Songli Shi

**Affiliations:** 1 First Affiliated Hospital of Baotou Medical College, Baotou, China; 2 Department of Pharmacy, Baotou Medical College, Inner Mongolia University of Science and Technology, Baotou, China

**Keywords:** cirrhosis, metabolic markers, serum metabolomics, UPLC-QTOF/MS, urine metabolomics

## Abstract

**Introduction:**

Liver cirrhosis is the terminal stage of chronic liver disease, which is marked by high morbidity and mortality in its advanced phases. Although liver biopsy still serves as the gold - standard diagnostic method, the detection of serum and urine metabolites holds great promise for the identification of cirrhosis.

**Methods:**

Untargeted metabolomics analysis was carried out using ultra - performance liquid chromatography coupled with quadrupole time - of - flight mass spectrometry (UPLC - QTOF/MS). We compared the serum and urine metabolic profiles between 30 healthy individuals and 28 liver cirrhosis patients to screen for biomarkers associated with liver cirrhosis.

**Results:**

A total of 55 endogenous metabolites showed dysregulation in serum, and 51 did so in urine. Four shared differential metabolites—glycoursodeoxycholic acid, urobilin, glycocholic acid, and urobilinogen—were identified in both biofluids. Pathway enrichment analysis revealed three co - regulated metabolic pathways: tryptophan metabolism, glycerophospholipid metabolism, and porphyrin metabolism (*p* < 0.05).

**Discussion:**

This study delineates the distinct metabolic signatures of cirrhosis and proposes a diagnostic strategy based on dual - biofluid analysis. The intersectional biomarkers and pathways elucidate the mechanisms linking bile acid homeostasis and hemoprotein catabolism to cirrhotic progression, offering a noninvasive approach for clinical detection.

## 1 Introduction

Chronic liver diseases (CLDs) affect approximately 800 million individuals worldwide, leading to roughly 2 million fatalities per year (1, 2). The progression of CLDs can be ascribed to a variety of factors, such as alcohol misuse, obesity and other metabolic disorders, autoimmune hepatitis, and viral hepatitis (3). Cirrhosis represents the end-stage consequence of chronic hepatitis and can advance to diffuse cirrhosis. In patients with cirrhosis, the normal hepatic architecture is supplanted by regenerative nodules, and in severe instances, it may culminate in liver failure. The early stage of cirrhosis is frequently asymptomatic yet potentially reversible (4). Initial assessments for cirrhosis encompass serological assays for viral hepatitis, quantification of ferritin and transferrin saturation, abdominal ultrasonography, a complete blood count, liver function evaluations, and determination of the prothrombin time/international normalized ratio (5). The therapeutic objectives for cirrhosis are to prevent its onset, decompensation, and mortality. Non-selective beta-blockers are frequently employed (6, 7). The management of ascites entails the use of diuretics, salt restriction, and antibiotic therapy. Liver biopsy still serves as the gold-standard method for the detection of cirrhosis. Non-invasive assessments are particularly valuable in identifying either the early or advanced stages of cirrhosis. Serum-based biomarkers for cirrhosis have been established (8).

Metabolomics, a crucial domain in “omics” research, is centered on the high-throughput identification and quantitative assessment of small-molecule metabolites (with a molecular weight < 1,500 Da) in the metabolome (9, 10). The progress of analytical technologies and bioinformatics has elevated metabolomics to a key position as a systems biology tool, propelling its extensive application as an integrated diagnostic strategy in clinical and biomedical research (11, 12). Yang (13) uncovered the distinctive metabolic features of cancer cachexia via serum and urine metabolomics and developed a diagnostic model. Blood, being a vital and easily accessible biofluid, is of great significance in clarifying the pathogenesis and progression of human diseases (14). Urine represents an optimal biofluid for disease research owing to its non-invasive collection and relatively simpler composition compared with other bodily fluids. The convenience of serial sampling facilitates the longitudinal monitoring of disease progression and the assessment of therapeutic response (15). In this study, untargeted metabolomics was utilized to characterize the serum and urine metabolites in patients with cirrhosis. Our findings provide mechanistic underpinnings for non-invasive diagnosis of cirrhosis and identification of therapeutic targets.

## 2 Materials and methods

### 2.1 Study participants

We recruited patients with cirrhosis from the First Affiliated Hospital of Baotou Medical College between February and July 2023, and obtained clinical data from 28 patients with cirrhosis. The diagnosis of cirrhosis included liver biopsy, imaging studies, coagulation tests, complete blood count, and complications of decompensated cirrhosis (ascites, gastrointestinal bleeding, sepsis, hepatic encephalopathy, and hepatorenal syndrome). The serum and urine samples of 30 healthy people without disease were obtained at the same time. Inclusion criteria of cirrhosis: (1) the cirrhosis group had clinical symptoms such as loss of appetite, anorexia, fatigue, and discomfort or pain in the liver region on admission; (2) the healthy group did not have any liver disease; (3) complete medical history, aged 18 years or older, and no cognitive impairment. Exclusion criteria include: (1) severe organic diseases; (2) diagnosis of liver cancer or other malignant tumors; (3) recent use of drugs that affect liver function indexes. The Medical Ethics Committee of the First Affiliated Hospital of Baotou Medical College approved this study (Ethical approval number: 2022026). Written informed consent was obtained from all participants. General data (age, gender, height, weight, body mass index, etc.) and laboratory examinations were collected for all subjects. All methods were performed in accordance with the relevant guidelines and regulations.

### 2.2 Untargeted metabolomics

The 100 μL sample was transferred to an eppendorf tube, and then 400 μL of extract (methanol: acetonitrile = 1:1) was added. The mixture was vortexed and mixed for 30s, and sonicated for 10 min under an ice water bath, and then left at −40 °C for 1 h. The serum samples were centrifuged at 12000 rpm for 15 min at 4 °C, and the supernatant was taken for detection. Equal volumes of supernatant from all samples were mixed to make quality control (QC) samples. The QC samples were scanned at intervals of 10 samples during the collection process. Systematic errors were corrected with the quality gap between QC samples. The target compounds were separated by chromatography using a Vanquish ultra-high performance liquid chromatographer (Thermo Fisher Scientific). The parameters for setting the liquid phase gradient are as follows: 0.0–0.8 min, 2%B; 0.8–2.8 min, 2–70%B; 2.8–5.3 min, 70–90%B; 5.3–5.9 min, 90–100% B; 5.9–7.5 min, 100% B; 7.5–7.6 min, 100–2% B; 7.6–10.0 min, 2% B. The flow rate was 0.3 mL/min. Phase A of liquid chromatography consisted of 25 mmol/L ammonium acetate and 25 mmol/L ammonia, and phase B consisted of acetonitrile. Sample plate temperature: 4 °C, injection volume: 2 μL. Primary and secondary mass spectrometry data were acquired using a mass spectrometer (Orbitrap Exploris 120) controlled by Xcalibur (V4.4, Thermo). After converting the raw data into mzXML format, peak identification, extraction, alignment, and integration were performed. When integrating standardized datasets, probabilistic quotient normalization (PQN) was applied to ensure inter-sample comparability and mitigate scaling variability (16). By comparing the spectral intensities of a sample with those of other samples within its neighborhood, the probability quotient was calculated and the relative intensity was adjusted. The kyoto encyclopedia of genes and genomes (KEGG) database and human metabolome database (HMDB) were used to annotate metabolites, and the molecular weight data (m/z) were matched with the data in the database. Metabolites with a mass difference of less than 10 ppm between the observed and database values were annotated, and the molecular formula of metabolites were further identified and validated through isotope distribution measurements. Serum metabolic profile analysis was performed for all metabolites. Metabolites of variable importance in projection (VIP) > 1 and *p* < 0.05 were screened to draw the volcano map. The differential metabolites were compared with the information on the HMDB website^1^ to identify endogenous metabolites that met the requirements, and a heat map of metabolite expression clusters was generated. The endogenous differential metabolites were plotted using the MetaboAnalyst.^2^ We screened metabolic pathways according to impact > 0.02 and raw *p* < 0.05, and observed the key metabolites that affect cirrhosis within these metabolic pathways.

### 2.3 Statistical analysis

IBM SPSS Statistics 26.0 software was used to process the data of patients in this study. The enumeration data were expressed as n/%, and we used the chi-square test for calculations. The measurement data were expressed as x ± s, and we used the *t* and *F* test for calculations. To mitigate false positives arising from multiple testing, all statistical tests were corrected for the false discovery rate using the Benjamini-Hochberg procedure.

## 3 Results

### 3.1 Changes in serum and urine metabolic profiles

The separation trends between groups were observed using score plots. PCA (Principal Component Analysis) and PLS- DA (Partial Least Squares-Discriminant Analysis) were performed on the metabolites of the healthy control group and the disease group. Meanwhile, 200 times of 7-fold cross-validation was carried out on the PLS-DA results to determine whether the model was overfitted (Table 1).

**Table 1:** Differential metabolites in the serum of patients with cirrhosis.

No.	Rt(min)	m/z	Formula	Metabolite	HMDB ID	VIP	*p* value	Trend
								DIS vs. CON
1	0.79	241.03	C_6_H_12_N_2_O_4_S_2_	L-cystine	HMDB0000192	1.00	0.01	up
2	4.30	498.29	C_26_H_45_NO_6_S	taurodeoxycholic acid	HMDB0000896	2.67	0.00	up
3	4.18	448.31	C_26_H_43_NO_5_	glycoursodeoxycholic acid	HMDB0000708	1.89	0.00	up
4	4.18	899.63	C_26_H_43_NO_5_	glycohyodeoxycholic acid	HMDB0304944	1.54	0.00	up
5	8.46	585.27	C_33_H_36_N_4_O_6_	bilirubin	HMDB0000054	1.23	0.00	up
6	3.17	182.05	C_8_H_9_NO_4_	4-pyridoxic acid	HMDB0000017	1.08	0.02	up
7	3.10	188.18	C_9_H_21_N_3_O	N1-acetylspermidine	HMDB0001276	1.50	0.00	up
8	4.60	591.32	C_33_H_42_N_4_O_6_	urobilin	HMDB0004160	1.69	0.00	up
9	3.9	464.30	C_26_H_43_NO_6_	glycocholic acid	HMDB0000138	2.10	0.00	up
10	4.10	498.29	C_26_H_45_NO_6_S	chenodeoxycholyltaurine	HMDB0242411	2.68	0.00	up
11	4.21	407.28	C_24_H_40_O_5_	allocholic acid	HMDB0000505	1.45	0.00	up
12	3.36	318.19	C_15_H_27_NO_6_	2,4-dimethylhexanedioylcarnitine	HMDB0241047	1.71	0.00	up
13	4.11	383.33	C_27_H_42_O	N-[(3a,5b,7b)-7-hydroxy-24-oxo-3-(sulfooxy)cholan-24-yl]-glycine	HMDB0002409	1.79	0.00	up
14	3.96	627.38	C_33_H_57_O_9_P	PA(10:0/20:4(5Z,8Z,11Z,13E)-OH(15S))	HMDB0262659	1.42	0.00	up
15	5.16	389.26	C_24_H_38_O_4_	12 alpha-hydroxy-3-oxo-5beta-cholan-24-oic acid	HMDB0062742	1.19	0.00	up
16	7.25	674.44	C_36_H_68_NO_8_P	PC(14:1(9Z)/14:1(9Z))	HMDB0007900	2.37	0.00	up
17	4.16	627.38	C_33_H_57_O_9_P	PA(10:0/20:4(5Z,8Z,10E,14Z)-OH(12S))	HMDB0262661	1.63	0.00	up
18	4.29	253.14	C_11_H_18_N_4_O_3_	histidylvaline	HMDB0028898	1.01	0.00	up
19	4.21	625.36	C_33_H_55_O_9_P	PA(10:0/20:4(5Z,8Z,11Z,13E) + =O(15))	HMDB0262691	1.38	0.00	up
20	4.18	531.30	C_27_H_48_O_8_S	5b-cyprinol sulfate	HMDB0006888	1.98	0.00	up
21	1.25	203.15	C_8_H_18_N_4_O_2_	asymmetric dimethylarginine	HMDB0001539	1.07	0.00	up
22	3.64	448.31	C_26_H_43_NO_6_	glutamic acid	HMDB0000148	2.01	0.00	up
23	3.97	589.30	C_33_H_42_N_4_O_6_	urobilinogen	HMDB0004158	1.99	0.00	up
24	3.95	462.27	C_**26**_H_**45**_NO_**7**_S	taurocholic acid	HMDB0000036	2.83	0.00	up
25	2.26	181.05	C_9_H_10_O_4_	hydroxyphenyllactic acid	HMDB0000755	1.05	0.00	up
26	6.14	431.31	C_27_H_42_O_4_	7 alpha-hydroxy-3-oxo-4-cholestenoate	HMDB0012458	1.10	0.00	up
27	4.11	471.24	C_24_H_40_O_7_S	chenodeoxycholic acid 3-sulfate	HMDB0002586	1.64	0.00	up
28	5.07	433.24	C_21_H_39_O_7_P	LysoPA(18:2(9Z,12Z)/0:0)	HMDB0007856	1.73	0.00	down
29	6.13	436.28	C_21_H_44_NO_6_P	LysoPE(P-16:0/0:0)	HMDB0011152	1.73	0.00	down
30	5.78	500.28	C_25_H_44_NO_7_P	LysoPE(20:4(8Z,11Z,14Z,17Z)/0:0)	HMDB0011518	1.01	0.00	down
31	3.53	277.16	C_15_H_22_N_2_O_3_	phenylalanylisoleucine	HMDB0028998	1.91	0.00	down
32	5.72	524.28	C_27_H_44_NO_7_P	LysoPE(22:6(4Z,7Z,10Z,13Z,16Z,19Z)/0:0)	HMDB0011526	1.02	0.00	down
33	0.73	146.05	C_5_H_9_NO_4_	L-4-hydroxyglutamate semialdehyde	HMDB0006556	1.62	0.00	down
34	3.29	160.08	C_10_H_12_N_2_O	serotonin	HMDB0000259	1.22	0.00	down
35	5.25	518.32	C_26_H_48_NO_7_P	LysoPC(18:3(9Z,12Z,15Z)/0:0)	HMDB0010388	1.50	0.00	down
36	5.82	570.35	C_30_H_52_NO_7_P	LysoPC(22:5(7Z,10Z,13Z,16Z,19Z)/0:0)	HMDB0010403	1.52	0.00	down
37	5.59	546.34	C_28_H_52_NO_7_P	LysoPC(20:3(8Z,11Z,14Z)/0:0)	HMDB0010394	1.68	0.00	down
38	5.05	313.24	C_18_H_34_O_4_	octadecanedioic acid	HMDB0000782	1.74	0.00	down
39	0.92	124.01	C_2_H_7_NO_3_S	taurine	HMDB0000251	1.06	0.00	down
40	3.59	313.15	C_18_H_20_N_2_O_3_	phenylalanylphenylalanine	HMDB0013302	1.82	0.00	down
41	0.78	280.09	C_8_H_21_NO_6_P^+^	glycerophosphocholine	HMDB0000086	2.23	0.00	down
42	5.18	468.31	C_22_H_46_NO_7_P	LysoPC(14:0/0:0)	HMDB0010379	1.73	0.00	down
43	0.86	132.08	C_4_H_6_N_2_O_2_	dihydrouracil	HMDB0000076	1.02	0.00	down
44	0.72	132.03	C_4_H_7_NO_4_	L-aspartic acid	HMDB0000191	1.78	0.00	down
45	6.04	496.34	C_**24**_H_**50**_NO_**7**_P	LysoPC(16:0/0:0)	HMDB0010382	1.68	0.00	down
46	6.42	480.34	C_24_H_50_NO_6_P	LysoPC(P-16:0/0:0)	HMDB0010407	1.68	0.00	down
47	3.82	120.08	C_8_H_11_NO	2-hydroxyphenethylamine	HMDB0001065	1.34	0.00	down
48	6.20	303.23	C_20_H_32_O_3_	8-HETE	HMDB0004679	2.09	0.00	down
49	7.75	304.24	C_20_H_32_O_2_	arachidonic acid	HMDB0001043	1.23	0.00	down
50	9.39	546.35	C_26_H_54_NO_7_P	LysoPC(0:0/18:0)	HMDB0011128	1.80	0.00	down
51	3.76	199.01	C_8_H_8_O_4_S	4-vinylphenol sulfate	HMDB0062775	1.77	0.00	down
52	3.59	120.08	C_10_H_12_N_2_O_3_	kynurenine	HMDB0000684	1.44	0.00	down
53	3.93	308.19	C_17_H_25_NO_4_	4-phenylbutanoylcarnitine	HMDB0241867	1.82	0.00	down
54	4.82	267.12	C_14_H_20_O_5_	3-carboxy-4-methyl-5-pentyl-2-furanpropanoic acid	HMDB0061643	1.77	0.00	down
55	3.50	454.29	C_21_H_44_NO_7_P	LysoPE(16:0/0:0)	HMDB0011503	1.93	0.00	down

**Figure 1:**
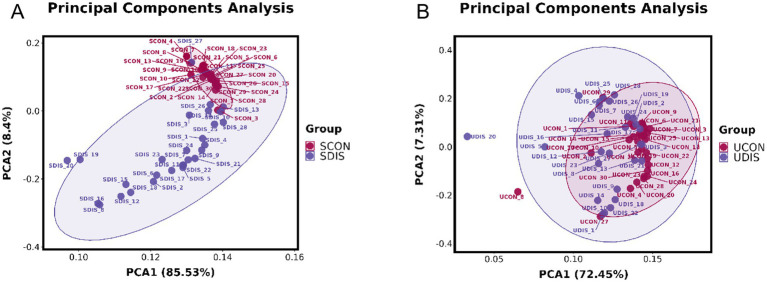
PCA score plots of serum and urine metabolomic profiles in patients with cirrhosis: **(A)** serum; **(B)** urine.

**Figure 2:**
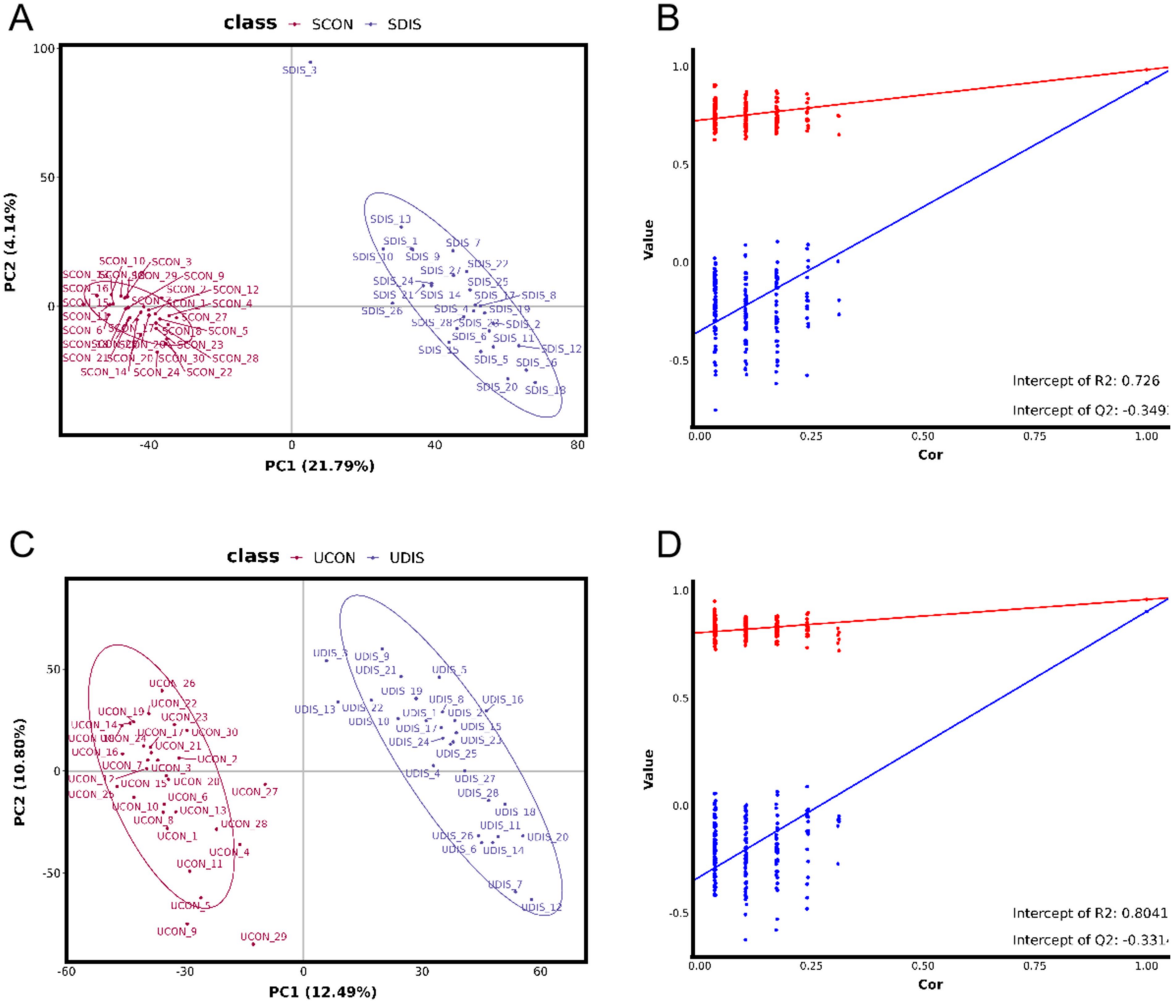
PLS-DA analysis of metabolomics in patients with cirrhosis: **(A)** PLS-DA score plot of serum samples; **(B)** Permutation test for the PLS-DA model of serum samples, R^2^ = 0.726, Q^2^ = −0.349; **(C)** PLS-DA score plot of urine samples; **(D)** Permutation test for the PLS-DA model of urine samples, R^2^ = 0.8041, Q^2^ = −0.331.

The PCA results showed that there was a significant deviation (8.4%) between the SCON group and the SDIS group along the PCA2 axis (Figure 1A). However, the difference between the UCON group and the UDIS group was not obvious (Figure 1B). The PLS-DA results indicated that there was a significant deviation (21.79%) between the SCON group and the SDIS group along the PC1 axis (Figure 2A). For the urine samples, there was also a significant deviation (12.49%) between the UCON group and the UDIS group along the PC1 axis (Figure 2C). Based on the permutation test results in panels B and D of Figure 2, the R^2^ and Q^2^ values of the original model are significantly higher than the distributions of the R^2^ and Q^2^ values of the permuted models. This indicates that there is a significant correlation between the grouping of samples (DIS vs. CON) and metabolites. It further supports our hypothesis that there are unique metabolic characteristics in cirrhosis patients (Figures 2B,D).

### 3.2 Screening of differential metabolites in serum and urine

**Figure 3:**
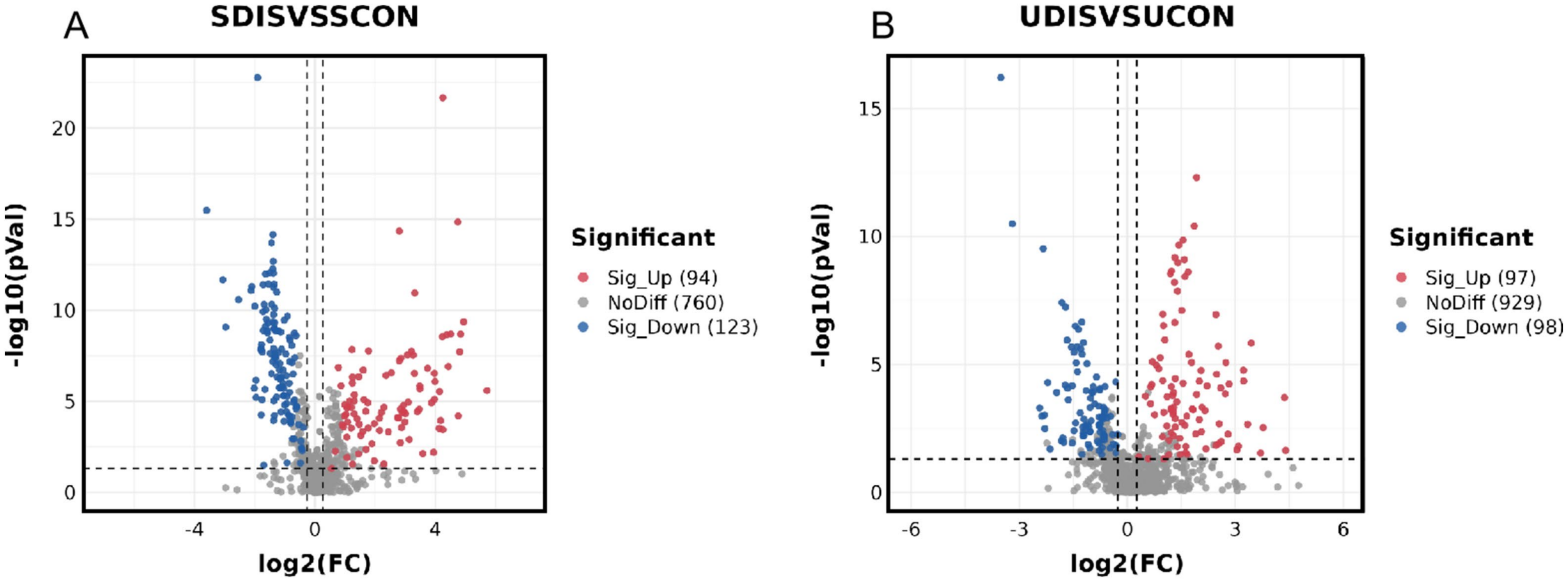
Metabolomics volcano map of patients with cirrhosis: **(A)** serum samples and **(B)** urine samples.

**Figure 4:**
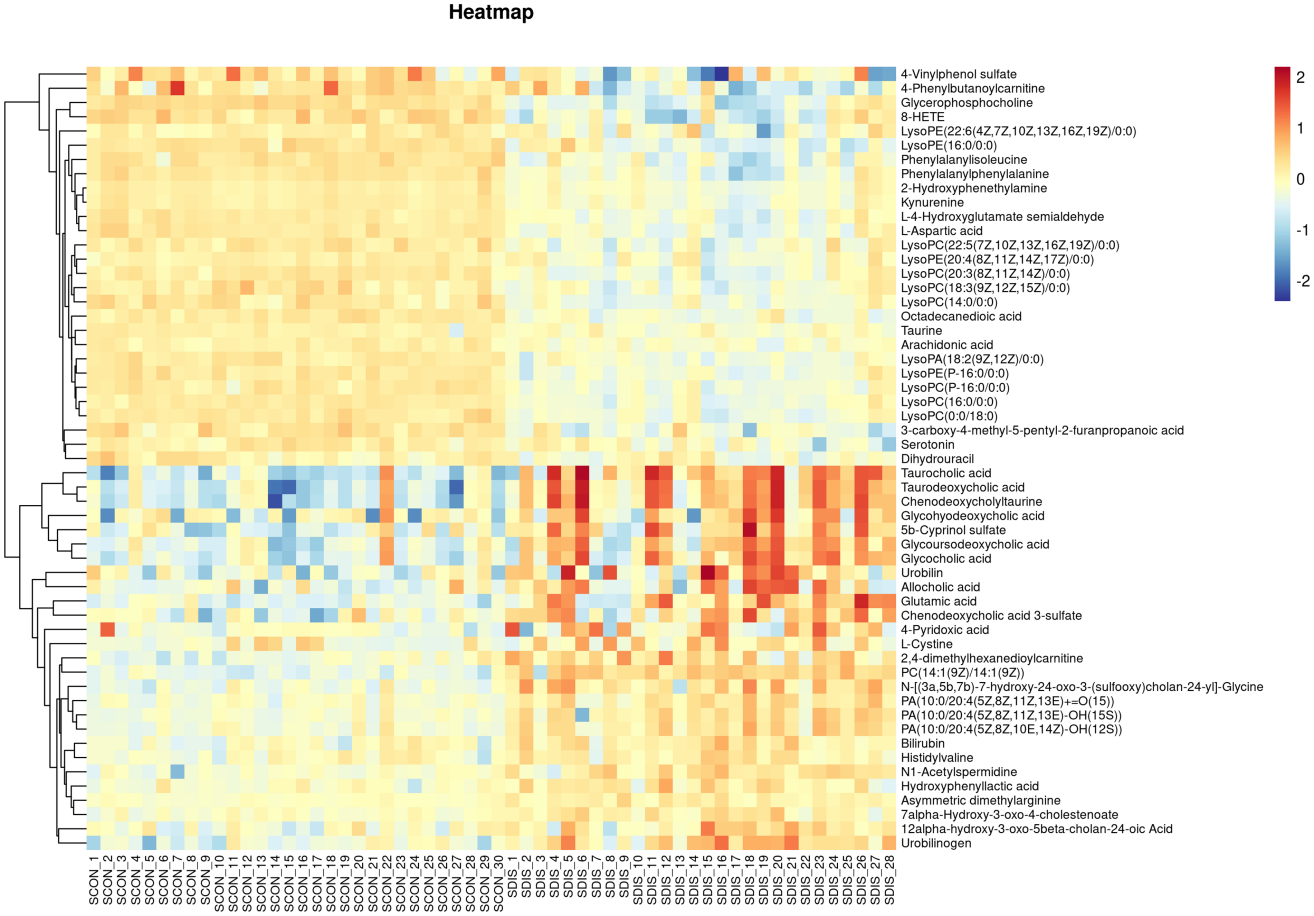
Heat map of metabolite expression in serum of patients with cirrhosis.

**Figure 5:**
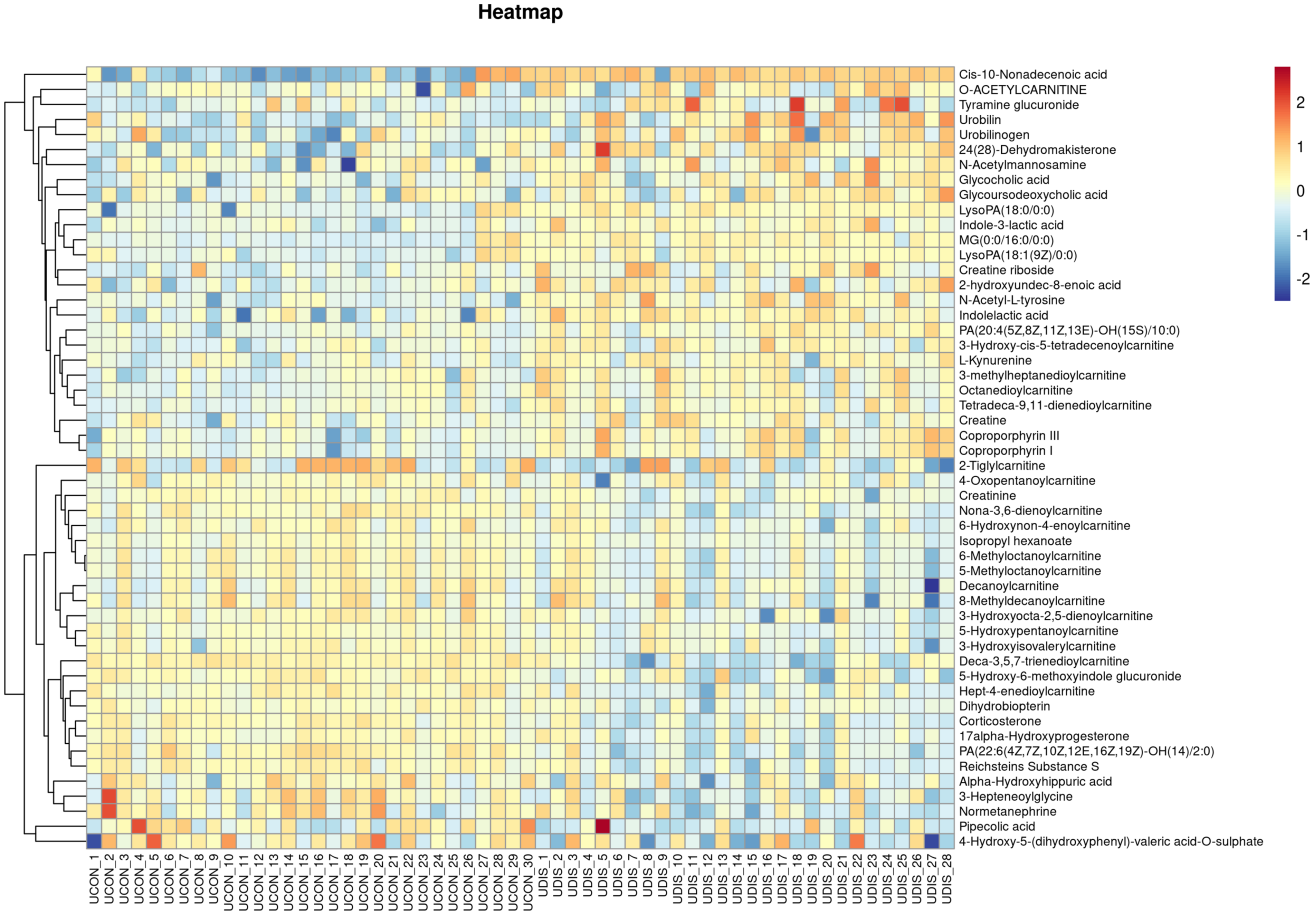
Heat map of metabolite expression in urine of patients with cirrhosis.

V-plots score graph was constructed with log_2_(FC) on the x-axis and -log_10_(*P*) on the y-axis for all metabolites to visualize the overall distribution of differential metabolites. Potential differential metabolites associated with the disease were screened with VIP > 1 and *p* < 0.05. Compared with the healthy control group, in the serum of the liver cirrhosis group, the contents of 94 metabolites were up-regulated, and those of 123 metabolites were down-regulated (Figure 3A). In urine, the contents of 97 metabolites were up-regulated, and those of 98 metabolites were down-regulated (Figure 3B). The metabolites obtained from the V-plots score graph were further screened on the HMDB website (see Footnote 1) to identify potential metabolic biomarkers associated with the disease. Heat maps of metabolite contents showed that 55 endogenous metabolites in the serum of liver cirrhosis patients were affected (Figure 4), and 51 endogenous metabolites in urine were affected (Figure 5). Among them, 4 metabolites were differentially affected in both serum and urine of liver cirrhosis patients: glycoursodeoxycholic acid, urobilin, glycocholic acid, and urobilinogen. The contents of these four metabolites were significantly up-regulated in the body fluid samples of liver cirrhosis patients (Table 2).

**Table 2:** Differential metabolites in the urine of patients with cirrhosis.

No.	Rt(min)	m/z	Formula	Metabolite	HMDB ID	VIP	*p* value	Trend
								DIS vs. CON
1	4.64	316.25	C_17_H_33_NO_4_	decanoylcarnitine	HMDB0000651	1.02	0.03	down
2	7.22	313.27	C_19_H_38_O_4_	MG(0:0/16:0/0:0)	HMDB0011533	1.63	0.00	up
3	4.41	302.23	C_16_H_31_NO_4_	6-methyloctanoylcarnitine	HMDB0241048	1.36	0.00	down
4	2.87	222.08	C_11_H_13_NO_4_	N-acetyl-L-tyrosine	HMDB0000866	1.93	0.00	up
5	4.58	330.26	C_18_H_35_NO_4_	8-methyldecanoylcarnitine	HMDB0240799	1.16	0.02	down
6	4.12	302.23	C_16_H_31_NO_4_	5-methyloctanoylcarnitine	HMDB0241049	1.43	0.00	down
7	4.22	591.32	C_33_H_42_N_4_O_6_	urobilin	HMDB0004160	2.02	0.00	up
8	4.52	655.28	C_36_H_38_N_4_O_8_	coproporphyrin III	HMDB0000570	1.66	0.00	up
9	4.29	655.28	C_36_H_38_N_4_O_8_	coproporphyrin I	HMDB0000643	1.67	0.00	up
10	2.59	209.09	C_10_H_12_N_2_O_3_	L-kynurenine	HMDB0000684	1.17	0.00	up
11	3.80	206.08	C_11_H_11_NO_3_	indole-3-lactic acid	HMDB0000671	1.43	0.00	up
12	1.43	204.13	C_9_H_18_NO_4_	o-acetylcarnitine	HMDB0000201	1.05	0.03	up
13	3.30	244.15	C_12_H_21_NO_4_	2-tiglylcarnitine	HMDB0241658	1.63	0.00	down
14	4.05	464.30	C_26_H_43_NO_6_	glycocholic acid	HMDB0000138	1.52	0.00	up
15	1.24	130.09	C_6_H_11_NO_2_	pipecolic acid	HMDB0000070	1.10	0.03	up
16	3.93	130.07	C_4_H_9_N_3_O_2_	creatine	HMDB0000064	1.03	0.01	up
17	1.67	262.16	C_12_H_23_NO_5_	5-hydroxypentanoylcarnitine	HMDB0241655	1.09	0.00	down
18	4.49	491.30	C_28_H_44_O_7_	24(28)-dehydromakisterone	HMDB0302988	2.02	0.00	up
19	3.55	318.19	C_15_H_27_NO_6_	3-methylheptanedioylcarnitine	HMDB0241046	1.28	0.00	up
20	3.66	340.18	C_17_H_25_NO_6_	deca-3,5,7-trienedioylcarnitine	HMDB0241129	2.36	0.00	down
21	2.55	302.16	C_14_H_23_NO_6_	hept-4-enedioylcarnitine	HMDB0241688	1.53	0.00	down
22	3.81	204.07	C_11_H_11_NO_3_	indolelactic acid	HMDB0000671	2.02	0.00	up
23	3.79	329.21	C_21_H_30_O_4_	corticosterone	HMDB0001547	1.75	0.00	down
24	2.53	194.05	C_9_H_9_NO_4_	alpha-hydroxyhippuric acid	HMDB0002404	1.06	0.02	down
25	0.85	264.12	C_9_H_17_N_3_O_6_	creatine riboside	HMDB0240254	1.44	0.00	up
26	1.51	314.12	C_14_H_19_NO_7_	tyramine glucuronide	HMDB0010328	1.12	0.03	up
27	7.89	297.28	C_19_H_36_O_2_	cis-10-nonadecenoic acid	HMDB0013622	3.32	0.00	up
28	3.35	318.19	C_15_H_27_NO_6_	octanedioylcarnitine	HMDB0241733	1.11	0.00	up
29	5.94	437.26	C_21_H_43_O_7_P	lysoPA(18:0/0:0)	HMDB0007854	1.63	0.00	up
30	3.74	539.25	C_27_H_41_O_9_P	PA(22:6(4Z,7Z,10Z,12E,16Z,19Z)-OH(14)/2:0)	HMDB0266564	2.18	0.00	down
31	3.96	627.37	C_33_H_57_O_9_P	PA(20:4(5Z,8Z,11Z,13E)-OH(15S)/10:0)	HMDB0262660	1.38	0.00	up
32	2.80	340.10	C_15_H_17_NO_8_	5-hydroxy-6-methoxyindole glucuronide	HMDB0010363	1.69	0.00	down
33	4.72	386.29	C_21_H_39_NO_5_	3-hydroxy-cis-5-tetradecenoylcarnitine	HMDB0013330	1.22	0.00	up
34	3.92	398.25	C_21_H_35_NO_6_	tetradeca-9,11-dienedioylcarnitine	HMDB0241391	1.20	0.00	up
35	4.01	329.21	C_21_H_30_O_4_	reichsteins Substance S	HMDB0000015	1.77	0.00	down
36	5.65	435.24	C_21_H_41_O_7_P	LysoPA(18:1(9Z)/0:0)	HMDB0007855	1.27	0.00	up
37	3.71	184.10	C_9_H_15_NO_3_	3-hepteneoylglycine	HMDB0094729	1.52	0.00	down
38	4.35	199.13	C_11_H_20_O_3_	2-hydroxyundec-8-enoic acid	HMDB0340607	1.38	0.00	up
39	1.62	260.15	C_12_H_21_NO_5_	4-oxopentanoylcarnitine	HMDB0241664	1.06	0.01	down
40	3.91	298.20	C_16_H_27_NO_4_	nona-3,6-dienoylcarnitine	HMDB0241762	1.82	0.00	down
41	4.13	157.12	C_9_H_18_O_2_	isopropyl hexanoate	HMDB0040430	1.01	0.00	down
42	3.31	305.03	C_11_H_14_O_8_S	4-hydroxy-5-(dihydroxyphenyl)-valeric acid-O-sulfate	HMDB0059978	1.36	0.02	down
43	2.03	262.16	C_12_H_23_NO_5_	3-hydroxyisovalerylcarnitine	HMDB0061189	1.06	0.01	down
44	3.97	593.33	C_33_H_42_N_4_O_6_	urobilinogen	HMDB0004158	1.53	0.00	up
45	3.69	316.21	C_16_H_29_NO_5_	6-hydroxynon-4-enoylcarnitine	HMDB0241753	1.06	0.01	down
46	1.56	240.11	C_9_H_13_N_5_O_3_	dihydrobiopterin	HMDB0000038	1.11	0.00	down
47	3.48	300.18	C_15_H_25_NO_5_	3-hydroxyocta-2,5-dienoylcarnitine	HMDB0241723	1.29	0.00	down
48	3.75	331.23	C_21_H_30_O_3_	17 alpha-Hydroxyprogesterone	HMDB0000374	1.21	0.00	down
49	4.27	414.30	C_26_H_43_NO_5_	glycoursodeoxycholic acid	HMDB0000708	1.12	0.02	up
50	4.10	204.09	C_8_H_15_NO_6_	N-acetylmannosamine	HMDB0001129	1.16	0.02	up
51	3.48	182.08	C_9_H_13_NO_3_	normetanephrine	HMDB0000819	1.43	0.00	down

### 3.3 Analysis of differential metabolic pathways in serum and urine

**Figure 6:**
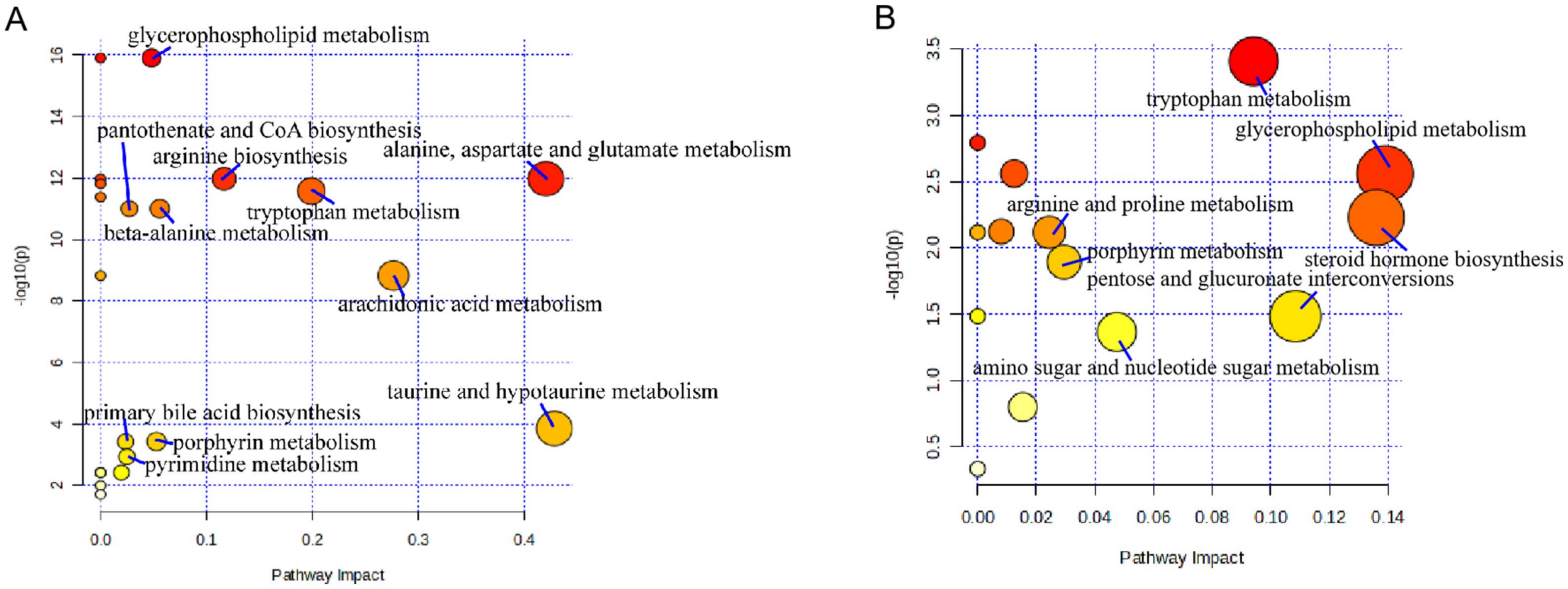
Metabolic pathways affected by patients with cirrhosis: **(A)** serum and **(B)** urine.

Pathway analysis of differential metabolites was performed on the MetaboAnalyst6.0 website (see Footnote 2). The key metabolic pathways influencing liver fibrosis were screened out based on raw *p* < 0.05 and impact > 0.02. It was found that liver cirrhosis affected 11 metabolic pathways (Figure 6A) in serum samples, including glycerophospholipid metabolism, alanine, aspartate and glutamate metabolism, arginine biosynthesis, tryptophan metabolism, beta-alanine metabolism, pantothenate and CoA biosynthesis, arachidonic acid metabolism, taurine and hypotaurine metabolism, porphyrin metabolism, primary bile acid biosynthesis, pyrimidine metabolism. Cirrhosis affects seven metabolic pathways (Figure 6B) in urine samples, includingarginine and proline metabolism, porphyrin metabolism, amino sugar and nucleotide sugar metabolism, tryptophan metabolism, pentose and glucuronate interconversions, steroid hormone biosynthesis, glycerophospholipid metabolism. Tryptophan metabolism, glycerophospholipid metabolism, and porphyrin metabolism were co-regulated by both serum and urine.

## 4 Discussion

The development and progression of liver diseases are intricately associated with individuals’ dietary patterns and lifestyles. Liver cirrhosis has multifaceted etiologies, predominantly stemming from hepatitis viruses, alcohol intake, and bile stasis. Portal hypertension and impaired liver function serve as critical indicators for the diagnosis of liver cirrhosis. The liver assumes a pivotal role in metabolism, and analyzing the serum and urine of patients with liver cirrhosis aids in the identification of metabolic biomarkers related to the condition. To enhance the accuracy and efficiency of our investigation, we implemented stringent participant selection criteria. Inclusion was restricted to patients with clinically diagnosed cirrhosis, while individuals with severe comorbidities or recent use of medications known to affect metabolic profiles were excluded. This rigorous screening protocol ensured cohort homogeneity, thereby allowing clear observation of cirrhosis-associated metabolic alterations despite the limited sample size (n = 28).

Our research findings indicate that in the serum of patients with liver cirrhosis, the levels of bilirubin, glycocholate, L-glutamate, urobilinogen, taurocholate, and 7*α*-hydroxy-3-oxo-4-cholestenoate are significantly upregulated, whereas those of serotonin, taurine, glycerophosphocholine, L-aspartate, arachidonate, kynurenine, and dihydrouracil are significantly downregulated. Moreover, glycoursodeoxycholic acid, urobilin, glycocholic acid, and urobilinogen are the metabolites that show significant increases in both the serum and urine of patients with liver cirrhosis. The liver plays an important role in metabolic processes. Serum samples from patients with cirrhosis were analyzed to find serum biomarkers associated with cirrhosis. Bilirubin in the blood is primarily produced by reticuloendothelial cells in the spleen (17). Bilirubin binds to human peroxisome proliferator-activated receptor α (PPAR α), which contributes to the reduction of hepatic fat accumulation and the alleviation of obesity and metabolic dysfunctions (18, 19). Nevertheless, excessively elevated bilirubin levels (>150 μM) may trigger the responses of pruritus receptors, and pruritus serves as the initial manifestation of cholestasis (20). The binding of bilirubin to albumin is frequently employed to predict the long-term prognosis of patients with hepatocellular carcinoma (21) and shows a significant correlation with the histological stage of patients with primary biliary cirrhosis (22). Bilirubin exhibits reactive oxygen species scavenging activity and immunosuppressive effects (23). However, an excessively high level of bilirubin may act as an indicator of cirrhosis.

The study found that alterations in the function of the glutamate-nitric oxide-cGMP pathway in cirrhosis can cause changes in the nervous system, giving rise to hepatic encephalopathy. In this process, nitric oxide activates soluble guanylate cyclase, leading to increased expression levels of nitric oxide in the cerebral cortex and thereby affecting the patients’ neurological function (24). The dysregulation of bile acid metabolism and its subsequent accumulation in the liver result in progressive liver injury and fibrosis. Cirrhosis can cause bile duct rupture, leading to bile acid leakage. Therefore, the accumulation of bile acids in the blood is associated with cirrhosis (25). Primary bile acids are synthesized within hepatocytes via cholesterol oxidation. Subsequently, they are conjugated with glycine or taurine and then excreted into the gallbladder by the bile salt export pump (26). Studies have found that taurocholate promotes the activation of hepatic stellate cells through the S1PR2/p38 MAPK/YAP signaling pathway (27). The levels of amino acids also changed significantly in patients with cirrhosis. Taurine and L-aspartate are non-essential amino acids, and their levels decreased significantly in patients with cirrhosis. Taurine is mainly synthesized in the liver and kidneys. It can reduce lipid peroxidation products, alleviate inflammation, and prevent calcium accumulation. The deficiency of taurine in hepatocytes leads to severe liver injury and triggers compensatory hepatocyte proliferation, which is closely related to the development of cirrhosis (28). L-ornithine L-aspartate has been utilized for the prevention and treatment of hepatic encephalopathy in cirrhotic patients (29).

Our study offers additional evidence indicating that the deficiency of L-aspartate is linked to the development of cirrhosis. Research has revealed that the level of tryptophan significantly increased in cirrhotic rats (30). Kynurenine, a product of tryptophan catabolism, is associated with signaling within the host microbiome, immune cell responses, and neuronal excitability. A decrease in the total activity of tryptophan 2,3-dioxygenase in liver tissue impedes the conversion of tryptophan to kynurenine. Consequently, a reduced level of kynurenine can serve as an indicator of cirrhosis. Serotonin induces the contraction and proliferation of smooth muscle cells and stimulates endothelial cells to release vasodilator substances. The level of serotonin is implicated in diseases such as hypertension, primary pulmonary hypertension, and cirrhosis (31). Our study further validates the close association between serotonin and the development of cirrhosis. Tryptophan metabolism, glycerophospholipid metabolism, and porphyrin metabolism were identified as cirrhosis-associated pathways detected in both serum and urine. Tryptophan metabolism is closely linked to gut microbiota. As an essential amino acid acquired exclusively through dietary intake, tryptophan plays a central role in metabolism. Within the gut, tryptophan is metabolized into 5-hydroxytryptamine (5-HT, serotonin), kynurenine, and various indole derivatives, demonstrating significant associations with the pathogenesis and progression of obesity, diabetes, non-alcoholic fatty liver disease, and atherosclerosis (32).

Serum and urine samples offer advantages such as simple operation, short processing time, good repeatability, and low cost, facilitating rapid disease diagnosis. However, in clinical applications, various factors that may affect the results need to be carefully taken into account. Before sample collection, health education for patients should be enhanced, and standardizing sample collection and storage procedures is essential. Moreover, it is necessary to improve the operational proficiency and comprehensive capabilities of laboratory technicians to eliminate the influence of subjective factors on test results. It should be noted that a limitation of this study is the absence of a validation dataset. Constrained by factors such as patient availability, geographical distribution, and ethical approval, we were unable to assemble an independent validation cohort. Although alternative measures like strict sample screening, quality control, and multiple-testing correction were implemented, the lack of external validation may affect the generalizability of our findings. Future research should aim to address this limitation by including larger, multi-center validation cohorts to enhance the robustness and clinical applicability of the results. In this study, metabolomics data were not employed to construct models for predicting the likelihood or severity of fibrosis. Despite conducting a detailed characterization of the urinary and serum metabolomes, we did not conduct further in-depth analysis of these datasets to establish predictive frameworks. The development of models using serum metabolomics alone, urine metabolomics alone, or integrated serum-urine data presents a significant opportunity for clinical translation. Accurately predicting fibrosis progression is of critical importance for early diagnosis and therapeutic intervention in clinical practice. Future studies should concentrate on leveraging these metabolomic profiles to develop prediction models with greater clinical utility. For instance, machine learning algorithms could be utilized to integrate multi-metabolite features from both serum and urine, and then establish robust fibrosis classifiers that can be validated in larger clinical cohorts. Simultaneously, this study did not take into account the metabolite ratios between serum and urine as potential indicators of disease status. Metabolite ratios may offer better pathophysiological insights than individual metabolite concentrations, as changes in relative abundance across biological matrices often reflect the underlying disease mechanisms. In specific pathological conditions, key metabolite ratios may show progressive changes that correlate with disease progression. Future research should prioritize exploring the relationships between serum-urine metabolite ratios and clinical disease states, which may potentially uncover novel diagnostic or prognostic biomarkers to guide therapeutic strategies.

## Data Availability

The original contributions presented in the study are included in the article/Supplementary material, further inquiries can be directed to the corresponding authors.

## Footnotes

1
https://hmdb.ca/

2
https://www.metaboanalyst.ca/
