# Supplementary material for: Serum-urine metabolic integration via UPLC-QTOF/MS uncovers shared pathway biomarkers for cirrhosis diagnosis
Source: Front Med (Lausanne). 2026 Feb 9;12:1646323. doi: 10.3389/fmed.2025.1646323 (PMC12926344; doi:10.3389/fmed.2025.1646323)
Supplement: Supplementary file 1 [file Table_1.DOCX]

**Supplementary Table Clinical Parameters in Healthy Controls and Patients with Cirrhosis**

| **Group** | **No.** | **ALT（U/L）** | **AST（U/L）** | **CHE（U/L）** | **ALP（U/L）** |
| --- | --- | --- | --- | --- | --- |
| CON | 1 | 30 | 25 | 11487 | 111 |
|  | 2 | 77 | 44 | 8424 | 98 |
|  | 3 | 27 | 20 | 7610 | 78 |
|  | 4 | 11 | 13 | 4708 | 45 |
|  | 5 | 68 | 25 | 4552 | 70 |
|  | 6 | 92 | 30 | 6987 | 56 |
|  | 7 | 40 | 23 | 10706 | 86 |
|  | 8 | 12 | 12 | 6644 | 78 |
|  | 9 | 39 | 30 | 9887 | 122 |
|  | 10 | 19 | 21 | 8726 | 65 |
|  | 11 | 33 | 20 | 11530 | 49 |
|  | 12 | 11 | 15 | 7751 | 44 |
|  | 13 | 20 | 24 | 4809 | 49 |
|  | 14 | 6 | 17 | 4957 | 61 |
|  | 15 | 66 | 32 | 12149 | 66 |
|  | 16 | 72 | 35 | 8861 | 77 |
|  | 17 | 15 | 18 | 5955 | 52 |
|  | 18 | 20 | 18 | 8000 | 71 |
|  | 19 | 20 | 20 | 8577 | 94 |
|  | 20 | 31 | 21 | 8224 | 55 |
|  | 21 | 14 | 17 | 9658 | 147 |
|  | 22 | 30 | 24 | 7377 | 65 |
|  | 23 | 6 | 16 | 5657 | 55 |
|  | 24 | 14 | 19 | 6574 | 110 |
|  | 25 | 10 | 16 | 8708 | 63 |
|  | 26 | 11 | 14 | 9229 | 65 |
|  | 27 | 5 | 14 | 5676 | 91 |
|  | 28 | 12 | 25 | 4579 | 46 |
|  | 29 | 9 | 16 | 7204 | 52 |
|  | 30 | 15 | 21 | 7014 | 97 |
| DIS | 1 | 55 | 78 | 4755 | 189 |
|  | 2 | 63 | 59 | 1711 | 193 |
|  | 3 | 26 | 24 | 8494 | 74 |
|  | 4 | 85 | 95 | 3744 | 614 |
|  | 5 | 41 | 67 | 2618 | 48 |
|  | 6 | 18 | 32 | 1496 | 93 |
|  | 7 | 23 | 29 | 4767 | 75 |
|  | 8 | 55 | 78 | 4755 | 189 |
|  | 9 | 28 | 85 | 4580 | 64 |
|  | 10 | 37 | 69 | 1948 | 166 |
|  | 11 | 26 | 25 | 4738 | 87 |
|  | 12 | 29 | 62 | 2279 | 189 |
|  | 13 | 11 | 14 | 3083 | 50 |
|  | 14 | 16 | 71 | 3274 | 126 |
|  | 15 | 17 | 38 | 2636 | 281 |
|  | 16 | 64 | 107 | 3697 | 575 |
|  | 17 | 52 | 91 | 6223 | 71 |
|  | 18 | 16 | 28 | 3116 | 153 |
|  | 19 | 17 | 24 | 4547 | 80 |
|  | 20 | 18 | 56 | 5451 | 126 |
|  | 21 | 19 | 17 | 9954 | 81 |
|  | 22 | 29 | 92 | 2361 | 102 |
|  | 23 | 28 | 58 | 4294 | 95 |
|  | 24 | 19 | 28 | 4027 | 98 |
|  | 25 | 20 | 33 | 4109 | 78 |
|  | 26 | 115 | 168 | 1499 | 114 |
|  | 27 | 25 | 33 | 4483 | 66 |
|  | 28 | 18 | 29 | 4821 | 141 |
